# Aflatoxin-Exposure of *Vibrio gazogenes* as a Novel System for the Generation of Aflatoxin Synthesis Inhibitors

**DOI:** 10.3389/fmicb.2016.00814

**Published:** 2016-06-03

**Authors:** Phani M. Gummadidala, Yung Pin Chen, Kevin R. Beauchesne, Kristen P. Miller, Chandrani Mitra, Nora Banaszek, Michelle Velez-Martinez, Peter D. R. Moeller, John L. Ferry, Alan W. Decho, Anindya Chanda

**Affiliations:** ^1^Department of Environmental Health Science, Arnold School of Public Health, University of South Carolina, ColumbiaSC, USA; ^2^National Ocean Service, Hollings Marine Laboratory, CharlestonSC, USA; ^3^Department of Chemistry and Biochemistry, University of South Carolina, ColumbiaSC, USA

**Keywords:** aflatoxins, *Vibrio gazogenes*, *Aspergillus parasiticus*, aflatoxin response metabolites (ARMs), natural products

## Abstract

Aflatoxin is a mycotoxin and a secondary metabolite, and the most potent known liver carcinogen that contaminates several important crops, and represents a significant threat to public health and the economy. Available approaches reported thus far have been insufficient to eliminate this threat, and therefore provide the rational to explore novel methods for preventing aflatoxin accumulation in the environment. Many terrestrial plants and microbes that share ecological niches and encounter the aflatoxin producers have the ability to synthesize compounds that inhibit aflatoxin synthesis. However, reports of natural aflatoxin inhibitors from marine ecosystem components that do not share ecological niches with the aflatoxin producers are rare. Here, we show that a non-pathogenic marine bacterium, *Vibrio gazogenes*, when exposed to low non-toxic doses of aflatoxin B_1_, demonstrates a shift in its metabolic output and synthesizes a metabolite fraction that inhibits aflatoxin synthesis without affecting hyphal growth in the model aflatoxin producer, *Aspergillus parasiticus.* The molecular mass of the predominant metabolite in this fraction was also different from the known prodigiosins, which are the known antifungal secondary metabolites synthesized by this *Vibrio*. Gene expression analyses using RT-PCR demonstrate that this metabolite fraction inhibits aflatoxin synthesis by down-regulating the expression of early-, middle-, and late- growth stage aflatoxin genes, the aflatoxin pathway regulator, *aflR* and one global regulator of secondary metabolism, *laeA*. Our study establishes a novel system for generation of aflatoxin synthesis inhibitors, and emphasizes the potential of the under-explored *Vibrio*’s silent genome for generating new modulators of fungal secondary metabolism.

## Introduction

Aflatoxins are a group of secondary metabolites that are synthesized primarily by food-borne fungi such as *Aspergillus parasiticus* and *Aspergillus flavus*. These *Aspergilli* contaminate a variety of economically important crops such as corn, wheat, peanuts, tree nuts, dried fruits, vegetables, and medicinal plants in tropical and subtropical areas worldwide (Trail et al., 1995; Bennett and Klich, 2003; Chanda et al., 2009; Georgianna and Payne, 2009). Aflatoxin B_1_ is the most potent liver carcinogen known and its contamination in food and feed is a significant risk factor of liver cancer risk in humans and animals (CAST, 2003; Liu and Wu, 2010). With liver carcinomas already being the third leading cause of cancer-related mortality worldwide, the global increase in prevalence of hepatitis B virus (HBV) and immunocompromised population has increased the risk of aflatoxin-induced liver cancer (Liu and Wu, 2010). The elimination of aflatoxin accumulation in food and feed, therefore, is of primary importance for reducing its global burden on public health and economy.

Common agricultural approaches used for prevention of aflatoxin contamination in crops include use of fungicides, biocontrol agents and fungi-resistant plants, crop rotation, choice of a plantation time that avoids the aflatoxin-conducive climatic conditions, and control of environmental factors during post-harvest (Kabak et al., 2006; Wu and Khlangwiset, 2010a,b; Cary et al., 2011). However, most of these strategies are expensive, time-consuming and have demonstrated limited success. To complement these conventional strategies, the use of compounds and extracts, collected from plants and microbes that share ecological niches with the aflatoxin producers, are becoming increasingly popular (Holmes et al., 2008). Examples of these natural compounds include a variety of naturally derived volatile compounds (Greene-McDowelle et al., 1999; Zeringue, 2000; Roze et al., 2004, 2007b, 2011). Despite the significant efforts in discovering aflatoxin biocontrol agents, over 55 billion people worldwide still suffer from uncontrolled exposure to aflatoxin (Strosnider et al., 2006), resulting in an *est*. 25,200 to 155,000 liver cancer cases globally (Liu and Wu, 2010). Chronic low-level exposure to aflatoxins and other carcinogenic mycotoxins remains a serious health threat in the USA (Kensler et al., 1992) and it is estimated that children in rural areas of the southern USA ingest ∼40 μg aflatoxin each day through contaminated food; a situation contributing to the significant rise in aflatoxin-induced liver cancer cases (Stoloff and Friedman, 1976; Van Rensburg, 1977). NIH statistics indicate that 16,600 new cases of aflatoxin-induced liver cancer annually in the USA (Kensler et al., 2011). Therefore, the aflatoxin monitoring programs and the destruction and/or decontamination of agricultural commodities, which are adopted to meet aflatoxin levels imposed by regulations from USA and Europe for food and feed, remain an expensive and time-consuming process. Hence, development of additional novel methodologies and compounds for aflatoxin elimination is essential.

*Vibrio gazogenes* is an estuarine Gram-negative bacterium that is well-known for its ability to synthesize industrially relevant proteins such as amylases and proteases (Ratcliffe et al., 1982) and bactericidal and fungicidal pigments, magnesidin A (Imamura et al., 1994), prodigiosins and cycloprodigiosins (Allen et al., 1983). Previous studies have also shown that random mutations in this bacterium with 1-methyl-3-nitro-l-nitrosoguanidine expanded its metabolic output and activated the synthesis of additional bactericidal prodigiosin-related pigments, norprodigiosin and propyl prodigiosin (Alihosseini et al., 2010). This prompted us to hypothesize that a portion of the bacterium’s metabolic potential remains silent under normal growth conditions, and can be activated by genetic and environmental perturbations. In this study, we conducted alterations of metabolism in *V. gazogenes* through exposures to non-toxic doses of the mycotoxin, aflatoxin. While aflatoxin B_1_ has been reported to bind to several probiotic bacteria (Kabak et al., 2009) and has also demonstrated the ability to alter bioluminescence responses in *Vibrio fischeri* (Li et al., 2011), there remains a lack of understanding on how interaction of aflatoxin B_1_ or other mycotoxins affect fundamental bacterial cell biology. To our surprise, aflatoxin exposure to *V. gazogenes* diminished prodigiosin release into the growth medium, but additionally resulted in the production of a new compound that demonstrated the ability to specifically inhibit aflatoxin synthesis in the model aflatoxin producer, *A. parasiticus.* Here we report the findings of this study. We establish a novel system for generation of aflatoxin-inhibitors and provide a new avenue in our fundamental understanding of *Vibrio* cell biology.

## Materials and Methods

### Ethics Statement

All experiments in this study were conducted in accordance with the general safety and biohazard protocols approved by the Envixronmental Health and Safety, University of South Carolina.

### Strains, Media, and Growth Conditions

*Aspergillus parasiticus*, SU-1 (ATCC 56775), a wild-type aflatoxin producer. The strain was grown on 100 mm Petri dishes containing potato dextrose agar for 2 weeks. Fresh spores collected from these colonies were used for all the experiments in this study that involved the use of SU-1. In these experiments the fungus was grown in aflatoxin-inducing yeast-extract-sucrose (YES); a rich growth medium (containing 2% w/v yeast extract, 6% w/v sucrose, pH 5.8), by inoculation of 10^4^ spores per mL of liquid medium and incubated in the dark (29°C; shaking at 150 rpm). A bacterial seawater isolate of *V. gazogenes*, having a 98% similarity of its 16S rRNA gene to ATCC 43942 (Farmer et al., 1988), was grown in Difco Marine Broth 2216 (BD Biosciences, Sparks, MD, USA) at 28°C in a shaking incubator (190 rpm). The bacterial isolate is currently being submitted to ATCC and NCBI.

### Growth Measurements of *A. parasiticus* and *V. gazogenes*

All fungal growth quantifications were performed using dry weight measurements. Briefly, the mycelia were filtered out of the growth media using a miracloth (Millipore, Billerica, MA, USA) and dried at 75°C for 6 h and the final weight was recorded. All *Vibrio* growth measurements were performed using absorbance readings of growth media at 600 nm.

### Aflatoxin Exposure Experiments, Extraction, and Analysis of *Vibrio* Metabolites

Aflatoxin B_1_ was commercially obtained (Sigma). Three different doses (0.1, 0.2, or 0.3 μg/mL) of aflatoxin B_1_ were added to the *Vibrio* growth medium at the start of the culture. In the control flask only the vehicle (70% Methanol) was added. To extract the metabolites from *V. gazogenes* the cells were first harvested by centrifugation and extracted with 60 mL acetone. A portion of the filtrate was concentrated by evaporation under N_2_ gas. The concentrate was loaded onto a silica gel column (1.2 cm × 15 cm) and eluted with dichloromethane: methanol (80:1.5). The fractions were then purified on a silica gel column using chloroform and methanol (50:2). After purification the fractions were concentrated by evaporation under N_2_ gas and re-suspended in 1 mL methanol for spectral analysis.

### ARMs Exposure Experiments and Aflatoxin Comparisons

Comparative semi-quantitative estimations of accumulation of aflatoxin in growth medium was performed using thin layer chromatography (TLC) of the growth medium as described previously (Banerjee et al., 2014).

### Total RNA Purification and Transcript Analysis

Isolation of total RNA from fungal cells exposed to aflatoxin response metabolites from *V. gazogenes* was performed using 30 h old cultures. This is a time point that corresponds to the activation of secondary metabolism (hence the expression of aflatoxin genes in *A. parasiticus*) under the growth conditions adopted in this study (Roze et al., 2007a). Purification of total RNA and preparation of complementary DNA was performed as described previously (Chanda et al., 2009). Transcript levels were quantified by performing quantitative real-time PCR assays using SsoAdvanced universal SYBR Green supermix (BioRad Laboratories, Hercules, CA, USA) and gene-specific forward and reverse primers (**Table 1**) that were designed using Primer3 online software^1^. Reactions were performed in a CFX96 thermal cycler (Bio-Rad Laboratories, Hercules, CA, USA). As described for previous gene expression studies in *A. parasiticus* (Roze et al., 2007a; Chanda et al., 2009), expression value of each gene was obtained from the threshold cycle values were normalized against β-tubulin (the house keeping gene) in each sample. All RT-PCRs were performed in triplicates for each gene per sample. Data analyses were performed using CFX Manager software (Bio-Rad Laboratories).

**Table 1:** List of PCR primers used for this study.

Genes	Primer sequences
(1) *nor-1*	F 5′-CACTTAGCCAGCACGATCAA-3′
	R 5′-ATGATCATCCGACTGCCTTC-3′
(2) *ver-1*	F 5′-AACACTCGTGGCCAGTTCTT-3′
	R 5′-ATATACTCCCGCGACACAGC-3′
(3) *β-tubulin*	F 5′-TCTCCAAGATCCGTGAGGAG-3′
	R 5′-TTCAGGTCACCGTAAGAGGG- 3′
(4) *aflR*	F 5′-ACCTCATGCTCATACCGAGG-3′
	R 5′-GAAGACAGGGTGCTTTGCTC- 3′
(5) *veA*	F 5′-TCCAGCTATCCCAAGAATGG-3′
	R 5′-TAATCCCCCGATAGAGCCTT-3′
(6) *laeA*	F 5′-ATGGGGTGTGGAAGTGTGAT-3′
	R 5′-ATCGGTAAAACCAGCCTCCT-3′

### Statistical Analyses

All statistical tests were performed using GraphPad Prism Software (GraphPad, La Jolla, CA, USA). Statistical analyses to determine for statistical significance of differences between control versus experimental groups were determined using one-way ANOVA (with sample size 3). An unpaired *t*-test was used to determine the gene expression effects of ARMs on *A. parasiticus* compared to the untreated samples. Significance was set at *p* < 0.05.

## Results

### Aflatoxin B_1_ Exposures Do Not Inhibit *V. gazogenes* Growth

**FIGURE 1:**
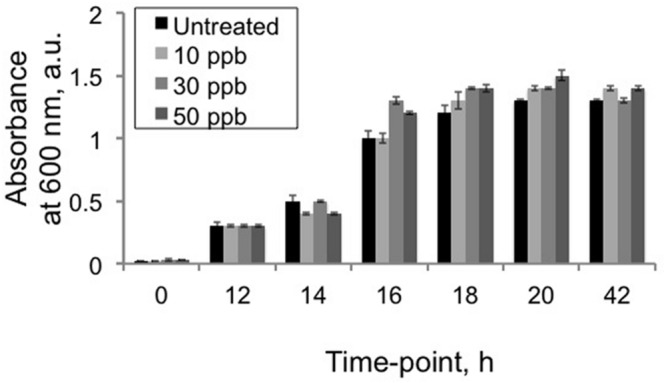
**Effect of aflatoxin B_1_ exposure on *Vibrio gazogenes* growth.** Growth comparisons were performed using comparisons of 600 nm absorbance values between untreated *V. gazogenes* cultures and cultures were supplemented with 10, 30, and 50 ppb of aflatoxin B_1_. Statistical significance of two-tailed *p*-values were determined using an unpaired *t*-test with sample size of 3 and significance set as *p* < 0.05.

As a first step in understanding how *V. gazogenes*, responds to aflatoxin B_1_, we investigated the effect of three different doses of aflatoxin B_1_ on the growth of *V. gazogenes.* The doses, 10, 30, and 50 ppb were either below, approximately equal to or fivefold higher than the highest-allowed aflatoxin level (20 ppb) in food and feed (Mazumder and Sasmal, 2001; CAST, 2003; Liu and Wu, 2010). Time-course absorbance readings were recorded to compare the growth rates of *V.* gazogenes, in presence of aflatoxin B_1_, with untreated-controls. As shown in **Figure 1**, none of the aflatoxin B_1_ doses demonstrated any significant effect on the growth of *V. gazogenes.*

### Aflatoxin B_1_ Exposures Do Not Inhibit Prodigiosin Synthesis

**FIGURE 2:**
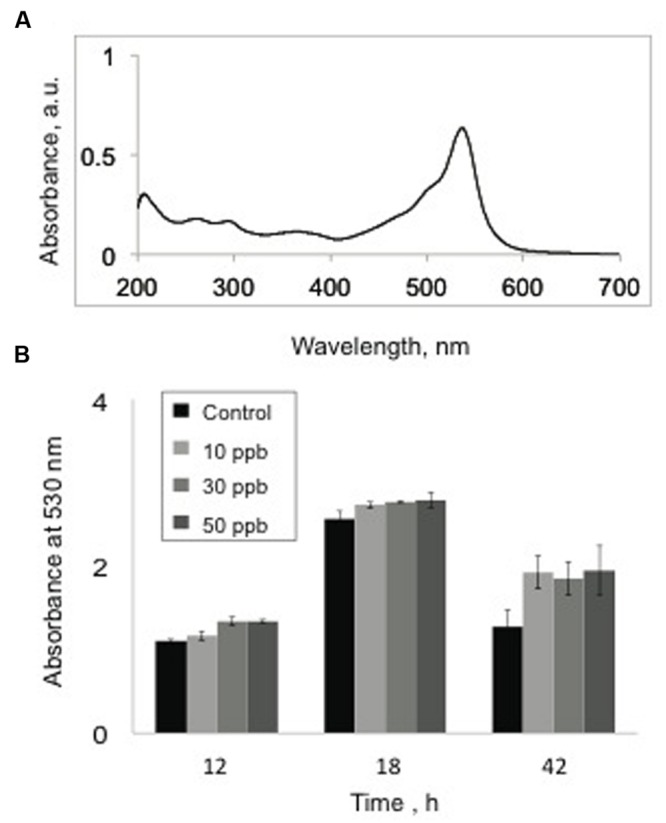
**Effect of aflatoxin B_1_ exposure on prodigiosin production. (A)** UV-Vis spectral profile of a prodigiosin-rich metabolite fraction demonstrating peak absorbance at 530 nm. **(B)** Comparison of absorbance values at 530 nm, of methanol extracts from untreated *V. gazogenes* cultures and cultures were supplemented with 10, 30, and 50 ppb of aflatoxin B_1_. Statistical significance of two-tailed *p*-values were determined using an unpaired *t*-test, with *n* = 3, and *p* < 0.05 as significance level.

Next, we investigated the effect of aflatoxin B_1_ exposures on the production of prodigiosins by *V. gazogenes*. The prodigiosin fraction was obtained from cells (either untreated control cells or cells exposed to aflatoxin B_1_) using our optimized laboratory protocol (see Materials and Methods). Since, the prodigiosins exhibit an absorbance peak at 530 nm (**Figure 2A**), this wavelength was used to compare prodigiosin levels between experimental treatments and controls at three different time-points of growth (12, 18, and 42 h). Our results (**Figure 2B**) demonstrated that although cells exposed to aflatoxin B_1_ showed a minor increase in absorbance values compared to the untreated samples, the difference was not statistically significant.

### Additional *V. gazogenes* Metabolite Fraction Obtained by Bacterial Exposure to Aflatoxin B_1_: Aflatoxin Response Metabolites (ARMs)

**FIGURE 3:**
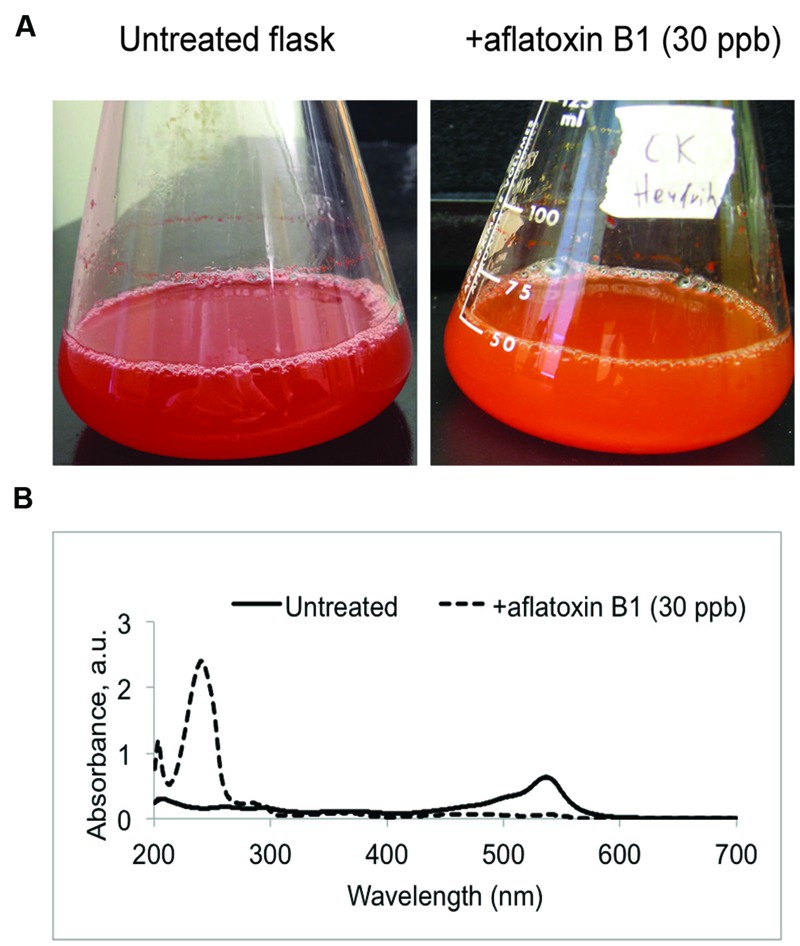
**Aflatoxin-response metabolites (ARMs) produced by the bacterium *V. gazogenes* during exposure to aflatoxin B_1_ (AFB1). (A)** Representative flasks demonstrating the differences in appearance of untreated *V. gazogenes* cultures and the aflatoxin B_1_ supplemented cultures. **(B)** Comparison of UV-Vis profiles of the methanol extracts from untreated and supplemented *V. gazogenes* cultures.

While growth and prodigiosin production by *V. gazogenes* was not affected in presence of aflatoxin B_1_, we observed that exposure to aflatoxin B_1_ resulted in a distinct alteration of color in the growth medium (**Figure 3A**) suggesting the presence of a different metabolite compared to untreated cells. Based on the ‘blue-shift’ in color of the growth medium (bright red to orange) upon addition of aflatoxin B_1_, we hypothesized that the bacterium synthesizes an additional metabolite fraction under these conditions with a corresponding absorbance lower than that of the prodigiosin fraction. To test this, we performed UV-Vis spectral analysis on the metabolite fractions of aflatoxin B_1_-treated samples. The *Vibrio* metabolite fractions obtained from aflatoxin B_1_ treated samples demonstrated a new absorbance peak at 240 nm (**Figure 3B**). This suggested that aflatoxin B_1_ exposure affects the cellular metabolism of *V. gazogenes* resulting in a different metabolite profile, compared to the untreated control. Here, we denote this additional metabolite fraction in response to aflatoxin B_1_ exposure as ‘aflatoxin response metabolites (ARMs).’

### ARMs Do Not Inhibit *A. parasiticus* Growth but Inhibit Aflatoxin Synthesis

**FIGURE 4:**
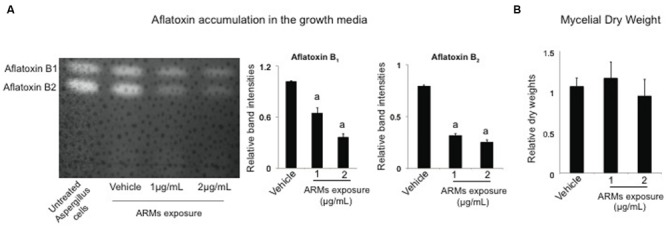
**Effect of ARMs on aflatoxin biosynthesis and fungal growth. (A)** Effect on aflatoxin accumulation in the growth media. Left panel, a representative TLC plate providing a qualitative comparison of aflatoxin accumulation in the untreated culture and cultures that were supplemented with 1 and 2 μg/mL ARMs extract and the vehicle (DMSO). Right panel, semi-quantitative comparative comparisons of band intensities of aflatoxin B_1_ and aflatoxin B_2_. a, significant difference in band intensity compared to the vehicle control. **(B)** Effect on growth. Comparison of dry-weight measurements. Bars represent measurements relative to the dry-weight of untreated cells. Statistical significance of two-tailed *p*-values were determined using an unpaired *t*-test, with sample size of *n* = 3 and *p* < 0.05 set as level of significance.

Next we proceeded to investigate whether ARMs affect the aflatoxin synthesis in the model aflatoxin B_1_ producer, *A. parasticus.* The activation of ARM production by *Vibrio* occurred upon addition of aflatoxin B_1_ to their growth medium. Therefore, we envisioned this alteration of metabolite profiles as a defensive response from *Vibrio* cells. We hypothesized that ARMs will have a specific inhibitory effect on aflatoxin synthesis in the producer cells. To test this we studied the growth and aflatoxin production by *A. parasiticus* in presence of two different doses of the ARMs metabolite fraction (1 and 2 μg per mL of growth medium); the doses were chosen arbitrarily. To compare the levels of aflatoxin biosynthesis in *A. parasiticus* exposed to ARMs exposed with the untreated cells, we adopted a semi-quantitative approach in which we compared the intensities of aflatoxin _1_ and aflatoxin B_2_ bands on the TLC plates (see Materials and Methods). As predicted, our TLC results generated from 40 h cultures of *A. parasiticus*, demonstrated that ARMs applied at the concentration of 2 μg per mL of growth medium inhibited both aflatoxin B_1_ and aflatoxin B_2_ by approximately twofold (**Figure 4A**). Since, the drop in aflatoxin synthesis could also have resulted from the inhibition of *A. parasiticus* growth, we next compared the dry-weights of the *A. parasiticus* mycelia exposed to 1 and 2 μg per mL of ARMs extract with the untreated control mycelia. As shown in **Figure 4B**, addition of ARMs to the growth medium did not result any significant change in *A. parasiticus* dry weight, suggesting that inhibition of aflatoxin synthesis in *A. parasiticus* by ARMs was a direct effect and not a growth dependent effect.

### ARMs Metabolite Fraction Displays a Different HPLC Trace Compared to Prodigiosin Fraction

**FIGURE 5:**
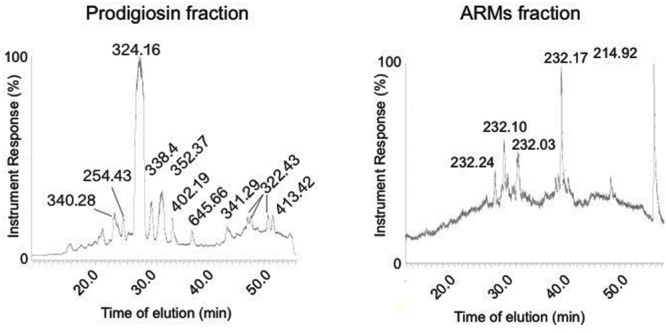
**Comparison of HPLC traces of ARMs extract and the prodigiosin fraction of *V. gazogenes***.

Our UV-Vis spectral analysis suggested that ARMs were synthesized by *V. gazogenes* upon exposure to aflatoxin. We then proceeded to confirm that this fraction (peak absorbance at 240 nm) was composed of metabolites of molecular masses that are different from the *Vibrio*’s prodigiosin fraction (peak absorbance at 530 nm). As shown in **Figure 5**, HPLC traces showed that the prodigiosin fraction predominantly demonstrated the expected molecular weight of 324 D, corresponding to the known prodigiosin. The HPLC trace of ARMs on the contrary was clearly different, with a predominantly displayed molecular mass 232 D, which demonstrate that the metabolite fraction of ARMs was chemically different from the *Vibrio’*s prodigiosin fraction. These results suggest that the differential metabolite profile in response to aflatoxin exposure can occur either due to synthesis of new metabolites by *V. gazogenes* or due to breakdown of prodigiosins resulting in novel smaller molecules with aflatoxin synthesis inhibitory activity.

### ARMs Inhibit *A. parasiticus* Aflatoxin Biosynthesis at the Level of Transcript Accumulation

**FIGURE 6:**
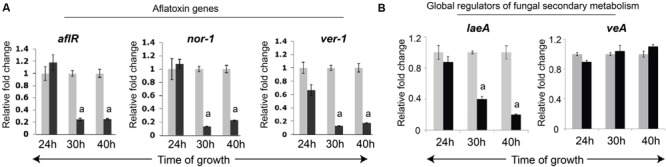
**Effects of ARMs on *Aspergillus parasiticus* gene expression. (A)** Comparison of transcript accumulation of aflatoxin-synthesis regulatory genes in *A. parasiticus*. mRNA levels for each gene were observed at 24 h (aflatoxin synthesis start point), 30 and 40 h time points (aflatoxin synthesis is activated and reaches peak levels by 40 h). Black bars, cells grown in presence of ARMs (2 μg/mL), Gray Bars, DMSO (vehicle) control. **(B)** Comparison of transcript accumulation of two global regulators of secondary metabolism, *veA* and *laeA* at the same time-points. Statistical significance of difference in transcript accumulation between control and ARMs-treated cells were determined using an unpaired *t*-test with sample size of 3 and two tailed *p* < 0.05 set as level of significance. a, *p* < 0.05.

The fungal growth and aflatoxin results then prompted us to investigate whether aflatoxin biosynthesis was inhibited at the level of transcript accumulation of aflatoxin genes. To conduct this analysis we performed a quantitative comparison of transcript accumulation of two genes *nor-*1, and *ver-1* that encode two enzymes, Nor-1, Vbs, and Ver-1, respectively, involved in the aflatoxin biosynthetic pathway (Chanda et al., 2009). Activation of these genes in *A. parasiticus* occurs at 24 h, and transcripts of all aflatoxin enzymes accumulate by 30 h, when the fungus is grown in YES growth medium (Roze et al., 2007a). Hence, we chose to examine the effects of ARMs extract on *A. parasiticus* at three different time-points, 24, 30 and 40 h, a time-point when aflatoxin is synthesized by the fungus at peak levels (Roze et al., 2007a). In addition to these genes, we also compared the transcript accumulation of the aflatoxin pathway regulator, *aflR*, at the same time points. As shown in **Figure 6**, *nor-1, ver-1* as well as the *aflR* genes transcript levels demonstrated ≥5 fold reduction in presence of ARMs extract compared to the vehicle control by 30 h. Hence, our results suggest that ARMs extract reduces aflatoxin synthesis at the level of transcript accumulation.

### ARMs Inhibit Transcript Accumulation of the Secondary Metabolism Global Regulator, *laeA* but not *veA*

**FIGURE 7:**
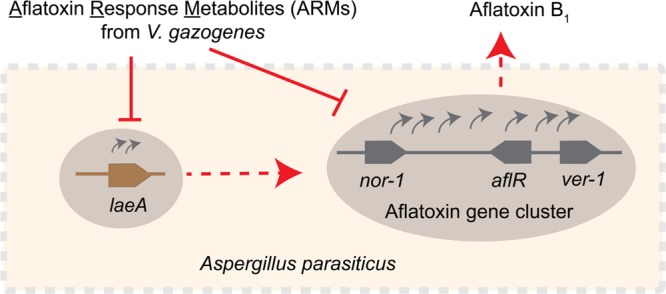
**Schematic representation of the inhibitory effect of ARMs on aflatoxin biosynthesis.** Current study demonstrates that ARMs inhibit aflatoxin biosynthesis in *A. parasiticus* at the level of gene expression. We hypothesize that the inhibition of aflatoxin genes as exemplified by the decreased *nor-1, ver-1*, and *aflR* transcripts in presence of ARMs can be the effect of one or both of the following: (1) inhibition of *laeA* expression, which in turn can have inhibitory impact on the activation of the aflatoxin genes, or (2) a dual inhibition caused by direct inhibition on aflatoxin gene cluster activation along with a *laeA* mediated inhibition. Red dotted arrows, regulatory roles established in previous studies, red solid lines, inhibitory effect, gray curved arrows, gene activation, gray solid line, schematic of the aflatoxin gene cluster showing relative positions of *nor-1, ver-1*, and *aflR* in the cluster, brown solid line, *laeA* gene.

Since the regulatory network of the aflatoxin biosynthesis pathway is integral to the global network of secondary metabolism in *A. parasiticus* (Chanda et al., 2009), we also proceeded to investigate whether, ARMs target the global regulation of secondary metabolism. One key global regulatory complex of fungal secondary metabolism is the *Velvet* complex which consists of proteins VeA, LaeA and other regulators (Bayram et al., 2008). Central to this complex is the cross-talk between the two global regulatory proteins, LaeA, a methyltransferase that is key to the epigenetic regulation of aflatoxin biosynthetic pathway (Bok and Keller, 2004), and VeA, a light responsive regulatory protein that migrates from cytoplasm to the nucleus in absence of light to particpate in the VeA complex (Bayram et al., 2008). In this study we investigated whether ARMs affect transcript accumulation of either *laeA* or *veA* genes. To our surprise we found that, while no significant changes occurred in *veA* transcripts, the *laeA* transcript accumulation was reduced by ∼2 fold by 30 h and ∼4 fold by 40 h (**Figure 7**), suggesting that ARMs inhibit aflatoxin biosynthesis at least in part, through inhibition of *laeA* gene activation.

## Discussion

Here, we demonstrate the feasibility of a novel system for generation of aflatoxin biosynthesis inhibitors, a concept that is analogous to the generation of antibodies upon antigen exposure. Our data reveal that the estuarine bacterium *V. gazogenes*, upon aflatoxin exposure, produces a metabolite profile that is chemically different from untreated-cells. Upon isolation of the ARMs and applying them on the aflatoxin producer cells, we found that the metabolites inhibit aflatoxin biosynthesis at the level of transcript accumulation of aflatoxin biosynthesis regulatory genes. Based on our current study we propose two possible explanations underlying this inhibition (illustrated in the schematic in **Figure 7**). One possible mechanism of inhibition is through the regulation of the *laeA* gene activation. The *laeA* transcripts dropped by 2–4 fold during 30–40 h time points suggesting that ARMs inhibit the formation of the *Velvet* complex, a protein complex comprising LaeA protein that regulate fungal secondary metabolism (Bayram et al., 2008). Alternatively, it is also possible that in addition to *laeA* mediated inhibition ARMs inhibit the activation of aflatoxin genes directly. Fungal growth was not inhibited during the ARMs-mediated inhibition of aflatoxin biosynthesis, suggesting that the metabolites target secondary metabolism specifically. Future studies will identify the molecule(s) within ARMs that results in the aflatoxin inhibition. From our current preliminary studies, we postulate that two or more compounds generated in response to aflatoxin exposure act either complementarily or synergistically to inhibit aflatoxin synthesis inhibition. These collaborative effects will be determined in those functional characterization studies with the purified compounds.

It is important to emphasize that specific aflatoxin inhibitory natural products that have been characterized to-date were reported primarily from terrestrial organisms whose ecological domains likely overlap with those of the aflatoxin producers. Examples include natural products and volatiles from plants (Cleveland et al., 2009; Roze et al., 2011; Chitarrini et al., 2014), fungi (Ono et al., 1997; Yoshinari et al., 2010; Hua et al., 2014) and bacteria (Jermnak et al., 2013; Wang et al., 2013; Kong et al., 2014). Our study provides the first evidence, to the best of our knowledge, of an organism that demonstrates the ability of synthesizing aflatoxin inhibitors, while not sharing ecological niches with aflatoxin producers at all. Also this is the first report, to the best of our knowledge, of a *Vibrio*-producing metabolite(s) that specifically inhibit aflatoxin biosynthesis without affecting fungal growth. It is possible that mycotoxin triggered synthesis of mycotoxin inhibitors is a phenomenon that is conserved in the *Vibrio* species. Alternatively, it is also possible that *V. gazogenes* is a chemically gifted organism that has genetically evolved with the rising mycotoxin levels in the environment with global changes in climate (Kolpin et al., 2014; Rangel et al., 2015).

The effect of ARMs mediated down-regulation of *laeA* gene, but not *veA* gene suggests that the metabolites target cellular signaling receptors that specifically regulate *laeA* gene expression. Since LaeA is a global regulator of secondary metabolism and influences several mycotoxin biosynthetic pathways (Keller et al., 2005), we anticipate that aflatoxin inhibitor within ARMs will inhibit other mycotoxins as well. Hence, for our follow-up studies we will categorize these as secondary metabolism specific inhibitors instead denoting these as specific inhibitors against aflatoxin biosynthesis.

Current investigations in our laboratory reveal that other fungal secondary metabolites trigger synthesis of metabolite fractions in *V. gazogenes* that demonstrate different HPLC traces compared to either prodigiosins or ARMs fractions. These results implicate the need to examine the regulation of *Vibrio* genes under different environmental signals. It appears from our studies that many areas of the *Vibrio* genome remain silent under standard laboratory growth conditions and can be activated as needed to generate metabolites that are relevant to the public health. Our future studies will shed light on these silent areas of the *V. gazogenes* genome that encode the biosynthesis of the secondary metabolism modulatory metabolites; the knowledge will enable us to clone these areas on plasmids and engineer them as needed with the goal of purifying these compounds in large quantities.

## Author Contributions

AC: conceived the research concept, PG, YPC, KB, KM, CM, NB, and MV-M: collected data, PG, YPC, PM, JF, AD, and AC: analyzed and interpreted the data and PG, AD, and AC: wrote the manuscript.

## Conflict of Interest Statement

The authors declare that the research was conducted in the absence of any commercial or financial relationships that could be construed as a potential conflict of interest.

## Footnotes

1 http://www.ncbi.nlm.nih.gov/tools/primer-blast/
